# Picroside II suppresses chondrocyte pyroptosis through MAPK/NF-κB/NLRP3 signaling pathway alleviates osteoarthritis

**DOI:** 10.1371/journal.pone.0308731

**Published:** 2024-08-29

**Authors:** Fanchen Wang, Jiacong Xiao, Miao Li, Qi He, Xintian Wang, Zhaofeng Pan, Shaocong Li, Haibin Wang, Chi Zhou

**Affiliations:** 1 First School of Clinical Medicine, Guangzhou University of Chinese Medicine, Guangzhou, China; 2 The Laboratory of Orthopedics and Traumatology of Lingnan Medical Research Center, Guangzhou University of Chinese Medicine, Guangzhou, China; 3 Department of Orthopedic Surgery, The First Affiliated Hospital, Guangzhou University of Chinese Medicine, Guangzhou, China; 4 Maoming Hospital of Guangzhou University of Chinese Medicine, Maoming, China; Shanghai Ninth People’s Hospital, Shanghai Jiao Tong University School of Medicine, CHINA

## Abstract

**Background:**

Picroside II (P-II) is the main bioactive constituent of Picrorhiza Kurroa, a traditional Chinese herb of interest for its proven anti-inflammatory properties. Its beneficial effects have been noted across several physiological systems, including the nervous, circulatory, and digestive, capable of treating a wide range of diseases. Nevertheless, the potential of Picroside II to treat osteoarthritis (OA) and the mechanisms behind its efficacy remain largely unexplored.

**Aim:**

This study aims to evaluate the efficacy of Picroside II in the treatment of osteoarthritis and its potential molecular mechanisms.

**Methods:**

In vitro, we induced cellular inflammation in chondrocytes with lipopolysaccharide (LPS) and subsequently treated with Picroside II to assess protective effect on chondrocyte. We employed the Cell Counting Kit-8 (CCK-8) assay to assess the impact of Picroside II on cell viability and select the optimal Picroside II concentration for subsequent experiments. We explored the effect of Picroside II on chondrocyte pyroptosis and its underlying molecular mechanisms by qRT-PCR, Western blot (WB) and immunofluorescence. In vivo, we established the destabilization of the medial meniscus surgery to create an OA mouse model. The therapeutic effects of Picroside II were then assessed through Micro-CT scanning, Hematoxylin-eosin (H&E) staining, Safranin O-Fast Green (S&F) staining, immunohistochemistry and immunofluorescence.

**Results:**

In in vitro studies, toluidine blue and CCK-8 results showed that a certain concentration of Picroside II had a restorative effect on the viability of chondrocytes inhibited by LPS. Picroside II notably suppressed the expression levels of caspase-1, IL-18, and IL-1β, which consequently led to the reduction of pyroptosis. Moreover, Picroside II was shown to decrease NLRP3 inflammasome activation, via the MAPK/NF-κB signaling pathway. In vivo studies have shown that Picroside II can effectively reduce subchondral bone destruction and osteophyte formation in the knee joint of mice after DMM surgery.

**Conclusions:**

Our research suggests that Picroside II can inhibit chondrocyte pyroptosis and ameliorate osteoarthritis progression by modulating the MAPK/NF-κB signaling pathway.

## Introduction

Osteoarthritis (OA) is a common chronic degenerative joint disease [1], its hallmark features include degeneration and damage to cartilage and the formation of bone spurs [2,3], they interfere with daily life and have a negative impact on physical and mental health [4,5]. The incidence of OA continues to rise as the population ages, and it is now one of the top three disabling diseases globally, alongside diabetes and dementia [6], posing a major challenge to public health.

Current therapeutic approaches to OA are predominantly medical and surgical. Medical treatment often involves the use of non-steroidal anti-inflammatory drugs (NSAIDs), acetaminophen, and corticosteroids [7–9]. While these medications can offer some pain relief, their efficacy is limited and they are associated with considerable side effects [10]. Surgical interventions, including total joint replacements, are considered the standard of care for advanced OA [11]. Nonetheless, the financial burden and the complexities of postoperative recovery following surgery present additional challenges [12]. Consequently, the quest for an efficient and safe novel treatment modality for OA remains a pivotal area of research focus.

There are various causes of OA, aging and obesity are currently recognized as the most important factors, others such as trauma and heredity are also part of the causes of OA [13]. The current study found that chondrocyte pyroptosis, contributes to the progression of OA [14]. Pyroptosis is alternatively known as inflammatory necrosis [15,16]. This process is predominantly dependent on the activation of inflammatory caspases, especially caspase-1, is marked by the emission of numerous pro-inflammatory cytokines [14]. Despite its biological importance, investigations that delineate the link between OA and pyroptosis are still limited in number [17]. The NLRP3 inflammasome, a complex with multiple functions, holds a significant role in the body’s innate immune response [18]. The complex is made up of the NLRP3 protein, ASC—which is an apoptosis-associated speck-like protein featuring a CARD—and pro-caspase-1, which is the precursor to caspase-1 [19]. In the context of OA pathogenesis, it is observed that Damage-associated molecular patterns (DAMPs) accumulate within the articular space [20]. These molecules initiate the activation of the caspase-1 and NLRP3 inflammasome [21], causing the release of IL-18 and IL-1β [22]. Remarkably, the levels of NLRP3 in individuals with osteoarthritis (OA) have been detected to exceed those in healthy subjects by a factor of more than five [23,24], this cascade of events leads to an elevation of inflammatory mediators within chondrocytes, intensifying the inflammatory response and propelling the disease’s progression [25,26]. Thus, focusing on chondrocyte pyroptosis may lead to new targets for the treatment of OA.

The MAPK/NF-κB signaling pathway significantly contributes to the pyroptotic demise of chondrocytes. For example, phosphorylation of JNK in the MAPK pathway activates the transcription factor c-Jun, and phosphorylation of p65 achieves elevation of NLRP3 by enhancing the transcriptional activity of NF-κB [27]. Meanwhile, the research by Zhou et al. has underscored the significance of the MAPK/NF-κB signaling pathway in inflammatory processes [28,29], which is a critical intracellular signaling mechanism regulating the inflammatory response and is essential for the activation of the NLRP3 inflammasome [30,31]. Hence, we posit that further investigation of the MAPK/NF-κB signaling pathway in the context of chondrocyte pyroptosis is warranted.

Picrorhiza Kurroa, a plant utilized in traditional Chinese medicinal practices, has been extensively used to address a range of ailments, including chronic fever, respiratory issues, and hepatic dysfunction [32–34]. P-II is a key active ingredient derived from this herb, which has been studied and shown to improve liver damage and protect heart muscle cells [35,36] also has shown promise in its ability to mitigate pyroptosis, anti-inflammatory and antioxidant [37]. Additionally, research has confirmed the potent anti-inflammatory effects of Picroside II, which are mediated through the regulation of the MAPK/NF-κB signaling pathway and the suppression of NLRP3 inflammasome activation. [38,39]. However, the potential of Picroside II in alleviating OA and the associated molecular mechanisms warrant additional study.

## Materials and methods

### Reagents

The primers listed in Table 1 were synthesized and acquired from Tsingke Biotechnology (Beijing, China). Evo M-MLV reverse transcription kit (AG11705) purchased from accurate biology (Hunan, China). p38, p65, JNK, ERK, p-ERK, p-JNK, p-p65 primary antibodies and secondary antibodies listed in Table 2 were obtained from Abmart Shanghai Co., Ltd. (Shanghai, China). In addition, MMP3, β-actin, β-tubulin were supplied by Affinity Biosciences, Inc. (Cincinnati, OH, USA), and Col2, NLRP3, IL-1β, caspase-1 were purchased from Wanleibio (Shenyang, China). IL-18 from Cell Signaling company of Technology (Beverly, MA, USA), p-p38 was provided by Thermo Fisher Scientific (Waltham, MA, USA). Picroside II (CAS 39012-20-9) was sourced from RuiFenSi Company, (Chengdu, China). Lipopolysaccharide (LPS) and collagenase type II were obtained from Sigma-Aldrich (Stuttgart, Germany). Servicebio Technology (Wuhan, China) offered toluidine blue Stain, foetal bovine serum (FBS, G8005), DMEM/F12 medium (G4610), 0.25% Trypsin Digestive Solution (G4001).

**Table 1:** qRT-PCR primer sequences.

Genes	Forward (5’-3’)	Reverse (5’-3’)
***18s***	**TGGTTGCAAAGCTGAAACTTAAAG**	**AGTCAAATTAAGCCGCAGGC**
***IL-1β***	**GGTGTGTGACGTTCCCATTA**	**ATTGAGGTGGAGAGCTTTCAG**
***NLRP3***	**CTGGCATCTGGGGAAACCT**	**GCTTCGGTCCACACAGAAAG**
***caspase-1***	**GAGAGAAAAGCCATGGCCGA**	**CCTTCACCCATGGAACGGAT**
***IL-18***	**AAGTAAGAGGACTGGCTGTG**	**CTCGGGTATTCTGTTATGGA**
***MMP3***	**CTCTGGAACCTGAGACATCACC**	**AGGAGTCCTGAGAGATTTGCGC**
***Col2***	**GCTGGTGAAGAAGGCAAACGAG**	**CCATCTTGACCTGGGAATCCAC**

**Table 2:** Primary antibody.

Name	Company	Concentration	Product number
**Col2**	**Wanleibio**	**1:2,000**	**WL03082**
**MMP3**	**Affinity Bioscience**	**1:2,000**	**AF0217**
**NLRP3**	**Wanleibio**	**1:1,000**	**WL02635**
**IL-1β**	**Wanleibio**	**1:2,000**	**WL00891**
**IL-18**	**Cell Signaling Technology**	**1:1,000**	**57058S**
**caspase-1**	**Wanleibio**	**1:2,000**	**WL03450**
**ERK**	**Abmart**	**1:1,000**	**T40071**
**JNK**	**Abmart**	**1:2,000**	**T40073**
**p-ERK**	**Abmart**	**1:1,000**	**TA1015**
**p-JNK**	**Abmart**	**1:1,000**	**T40074**
**p38**	**Abmart**	**1:1,000**	**T55488**
**p-p38**	**Thermofisher scientific**	**1:2,000**	**MA5-15218**
**p65**	**Abmart**	**1:5,000**	**T55034**
**p-p65**	**Abmart**	**1:1,000**	**TP56372**
**β-actin**	**Affinity Bioscience**	**1:1,500**	**T0022**
**β-tubulin**	**Affinity Bioscience**	**1:1,500**	**T0023**

### Cell culture

Chondrocytes were extracted from the knee joint cartilage of 7-day-old C57BL/6J mice. The cartilage was carefully minced, cartilage was first treated with a 0.25% trypsin solution for 30 minutes at a temperature of 37°C, followed by a 6 h digestion with a 0.1% collagenase solution at 37°C in a cell culture incubator. Then we Centrifuged and collected the cells. Chondrocytes were cultured at 37°C in a cell culture incubator using DMEM/F12 medium containing 10% FBS to be used for subsequent experiments [40].

### Cell viability assay

The impact of Picroside II on the viability of murine chondrocytes was assessed using the CCK-8 assay. Chondrocytes were seeded into a 96-well plate at a concentration of 1×10^3^ cells per well. Then we treated with different concentrations of P-II (5, 10, 25, 50, 100, 200μM) for 24 h in the condition with or without LPS ((1μg/mL) [41]. After treatment, the cells were incubate with 10μL of CCK-8 solution per well. Followed by a further 1 h incubation period. Subsequently, the absorbance of each well was determined at a wavelength of 450nm using a microplate reader.

### Toluidine blue staining

A total of 3×10^4^ chondrocytes were seeded into 24-well plates. The cells were subsequently exposed to LPS and varying concentrations of Picroside II (25μM and 50μM) for 24 h. Following this treatment, the cells were rinsed with PBS for 3 times. After that, the cells were incubated with a 4% paraformaldehyde solution for 15 minutes. Subsequently, the cells were stained with toluidine blue solution, and the cellular morphology was examined under a light microscope.

### Cellular immunofluorescence

Inoculate 1×10^5^ chondrocytes in a 24-well plate. After 24 h of treatment, the cells were rinsed with PBS. Subsequently, the cells were fixed with 4% paraformaldehyde solution. Cell membranes were permeabilized with 3% Triton X-100, afterward, the cells were blocked with a 3% BSA solution. Primary antibodies against NLRP3 and Col2(all at a dilution of 1:200) were applied and incubated at 4°C overnight, following that, the secondary antibody was introduced and the incubation was continued for 1.5 hours under light-protected conditions. Following 3 rinses with PBS, the cell nuclei were marked with DAPI stain. The cells were then examined using a fluorescence microscope.

### qRT-PCR

Extraction of total RNA using Trizol reagent and chloroform, centrifugation at 4°C. The RNA was then purified. Next, we measured the concentration, apply Evo M-MLV reverse transcription kit and follow the steps to complete the reverse transcription. mRNA levels were quantified using the Bio-Rad CFX96 system with SYBR Green I Master.

### Western blot

Cells were collected after the intervention. Proteins were isolated from chondrocytes by centrifugation at 4°C using a RIPA lysis buffer. The concentration of the extracted protein in each sample was then quantified utilizing a BCA (Bicinchoninic Acid) assay kit. Subsequently, the protein samples were combined with Loading buffer. The prepared samples were loaded onto a 10% SDS-PAGE gel and subsequently transferred to a PVDF membrane. The PVDF membrane was blocked with 5% BSA solution for 1.5 h. Then the membrane was then incubated with primary antibody at 4°C overnight, after which the membrane was thoroughly rinsed with TBST. It was then incubated with the secondary antibody for 1.5 h. Finally, protein bands were detected using an ECL imager, and the intensities of these bands were measured and quantified using ImageJ software.

### Animals experiment

Male C57BL/6J mice at 8-weeks-old were procured from the Animal Experimental Center at Guangzhou University of Chinese Medicine. Mice were anesthetized by intraperitoneal injection of 3% sodium pentobarbital (30 mg/kg). Mice excluding the control group underwent DMM surgery to induce an OA model. They were then allocated into groups: control (n = 8), model (n = 8). Based on previous research [42], we selected P-II low dose (P-II L, 25mg/kg, n = 8), and P-II high dose (P-II H, 50mg/kg, n = 8). Two doses of P-II were administered orally, and equal amounts of saline were given to the control and model groups. The mice were accommodated in a controlled environment, after 8 weeks of treatment, euthanasia of mice by overdose of sodium pentobarbital (200 mg/kg), after determining death, their knee joint of the hind limbs are ready to undergo the next experiment. All experimental protocols were thoroughly reviewed and were approved by the Ethics Committee of the First Affiliated Hospital of Guangzhou University of Traditional Chinese Medicine (GZTCMF1-20221121, Nov. 21,2022).

### Micro-CT scanning

The knee joints of mice were cleared of muscle tissue, and bone tissue was placed in 4% paraformaldehyde fixed for 24 h. Micro-CT imaging was conducted to scan the knee joints (scanning parameters: voltage 80kV, current 100μA, rotation step 0.4°, section thickness 5μm.), followed by 3D reconstruction using Skyscan CTvox software. Analysis programs (CTan, Skyscan) were used to calculate the parameters of the subchondral bone of the mouse knee joints, including bone volume fraction (BV/TV), trabecular number (Tb.N), trabecular thickness (Tb.Th), trabecular separation (Tb.Sp).

### Histological analysis

After the knee joints bone tissue was fixed with 4% paraformaldehyde, it was decalcified using 14% EDTA solution at 37°C for two weeks. Subsequently, the treated tissues underwent processing, embedding, and sectioning to prepare them for staining with H&E as well as S&F. The resultant slides were then examined using the Panoramic Midi digital slide scanner. The subsequent analysis of the stained slides was performed utilizing CaseViewer 2.4 software.

### Tissue immunofluorescence

Tissue sections were selected and initially deparaffinized using xylene after incubation in a 39°C oven. Subsequent sequential processing in a descending gradient of anhydrous ethanol solution was followed by application of pepsin repair solution to promote antigen repair. Using a histochemical pen, the area of interest on the slide was then depicted. A 3% BSA solution was uniformly applied to cover the tissue, primary antibodies targeting NLRP3 and MMP3 were added, and the slides were incubated at 4°C overnight. The secondary antibody was added dropwise the next day. The slides were then incubated at room temperature away from light. Final DAPI staining was performed to visualize the nuclei. The slides are then sealed with a coverslip and fluorescence is assessed using a fluorescence microscope.

### Immunohistochemistry

The tissue sections underwent a series of treatments starting with xylene and a gradient of anhydrous ethanol. They were then incubated with sodium citrate buffer. Subsequently, 0.1% trypsin was applied, the sections were blocked with 10% goat serum. Primary antibodies specific for NLRP3 and caspase-1 were added, Then, secondary antibodies were applied, the colorimetric development was performed using a DAB chromogen solution to visualize the antibody binding sites. The slides were then examined under a microscope to assess the immunohistochemical staining results.

### Statistical analyses

Each of the above experiments was repeated at least 3 times and the data were subjected to analysis utilizing SPSS 25.0 software, with results presented as the mean ± standard deviation. Comparisons between samples from multiple groups were made using one-way analysis of variance (ANOVO). *P* < 0.05 was deemed to indicate statistical significance. Additionally, Data visualization was accomplished through the use of GraphPad Prism 8.4.3 to generate graphical representations.

## Results

### Effects of P-II on cell viability in chondrocytes

**Fig 1:**
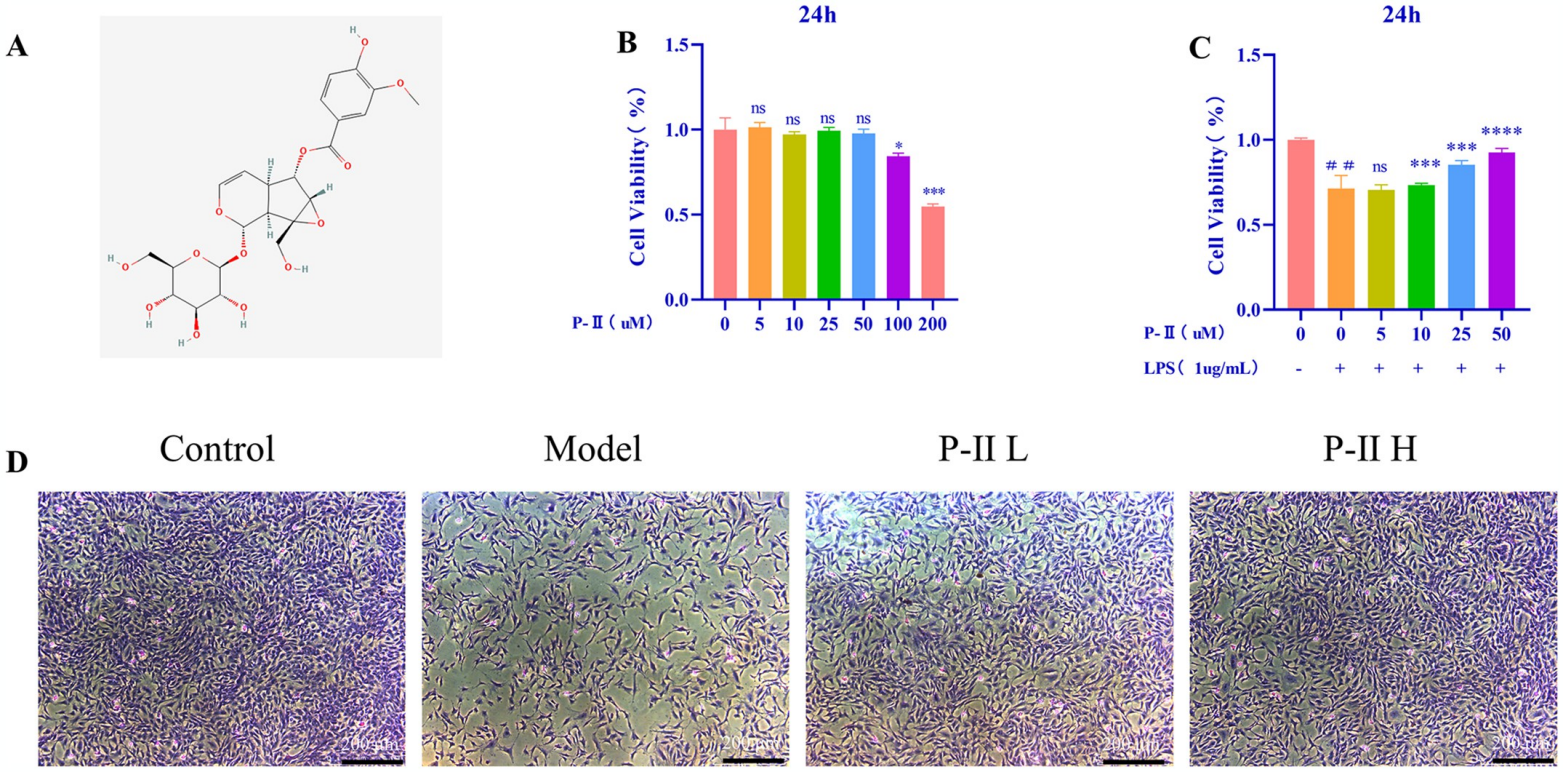
P-II counteracts the suppressive effect of LPS on chondrocyte proliferation. (A) Depicted is the chemical structure of Picroside II (P-II). (B) The impact of various concentrations of P-II (5, 10, 25, 50, 100, 200μM) on cell viability after a 24-hour incubation period is illustrated. (C) Shown is the effect of P-II (at concentrations of 5, 10, 25, 50μM) on cell viability over 24 hours under conditions with and without the addition of Lipopolysaccharide (LPS). (D) Representative picture of chondrocytes after toluidine blue staining. Data are presented as mean ± standard deviation (SD). Statistical significance is denoted by asterisks (* *P* < 0.05, ** *P*< 0.01, *** *P* < 0.001) and the symbol ## indicates *P* < 0.01, ns signifies no statistical significance.

In this study, we detected significant inhibition of chondrocyte viability at concentrations of P-II of 100μM and above. Subsequently, we established an inflammatory model in chondrocytes using LPS (1μg/mL). Surprisingly, P-II treatment resulted in a dose-dependent increase in cell viability, with 50μM P-II showing the most pronounced protective effect. At concentrations above 50μM, P-II exhibited inhibitory effects on cell proliferation. Consequently, we selected 25μM and 50μM P-II for subsequent experiments (Fig 1B and 1C). Toluidine blue staining revealed that P-II partially restored LPS-induced morphological changes in chondrocytes. (Fig 1D).

### P-II alleviates LPS induced ECM (Extracellular Matrix) degradation in chondrocytes

**Fig 2:**
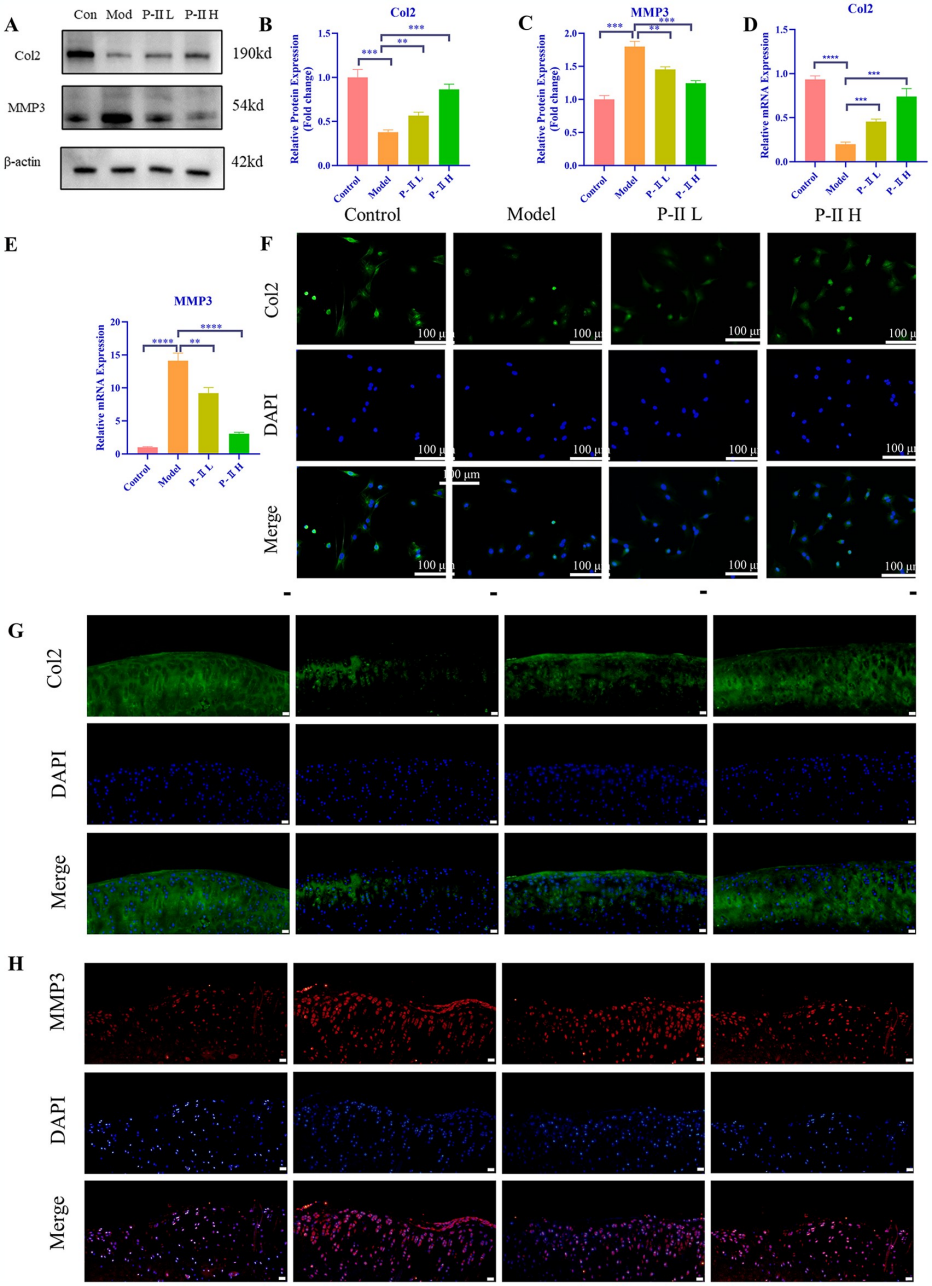
P-II attenuates ECM degradation in chondrocytes. (A) Representative Western blot images for Col2 and MMP3. (B, C) Quantitative analysis of the Western blot results for Col2 and MMP3. (D, E) qRT-PCR for *Col2* and *MMP3* gene expression. (F) Immunofluorescence Col2 (green) and dapi staining nuclear fluorescence (blue) are visible under fluorescence microscope. (G, H) Expression levels of Col2 and MMP3 in chondrocytes by immunofluorescence assay (Scale bar = 50μm). Data are presented as mean ± standard deviation (SD). * *P* < 0.05, ** *P* < 0.01, *** *P* < 0.001, **** *P* < 0.0001, ns signifies no statistical significance.

The ECM is a three-dimensional network structure composed primarily of Col2, and these components provide the necessary structural and mechanical support for cartilage [43]. According to the qRT-PCR and Western Blot analysis, we found that LPS decreased the expression of Col2 and increased the MMP3 levels at both the protein levels and mRNA (Fig 2A–2E). Meanwhile, we also confirmed the expression of Col2 in cellular immunofluorescence (Fig 2F) and tissue immunofluorescence consistent with the above experiments (Fig 2G). Similarly, the expression of MMP3 in tissue immunofluorescence was higher in the model group compared to the control group and decreased after treatment with P-II (Fig 2H) These results suggested that treatment with 25μM and 50μM P-II partially reversed these adverse LPS effects on chondrocytes. P-II treatment may play a role in restoring cartilage tissue. This suggests that P-II may have some favorable effects in promoting ECM repair and inhibiting ECM degradation, thereby slowing the progression of osteoarthritis.

### P-II suppresses NLRP3 inflammasome activation and alleviates chondrocyte pyroptosis

**Fig 3:**
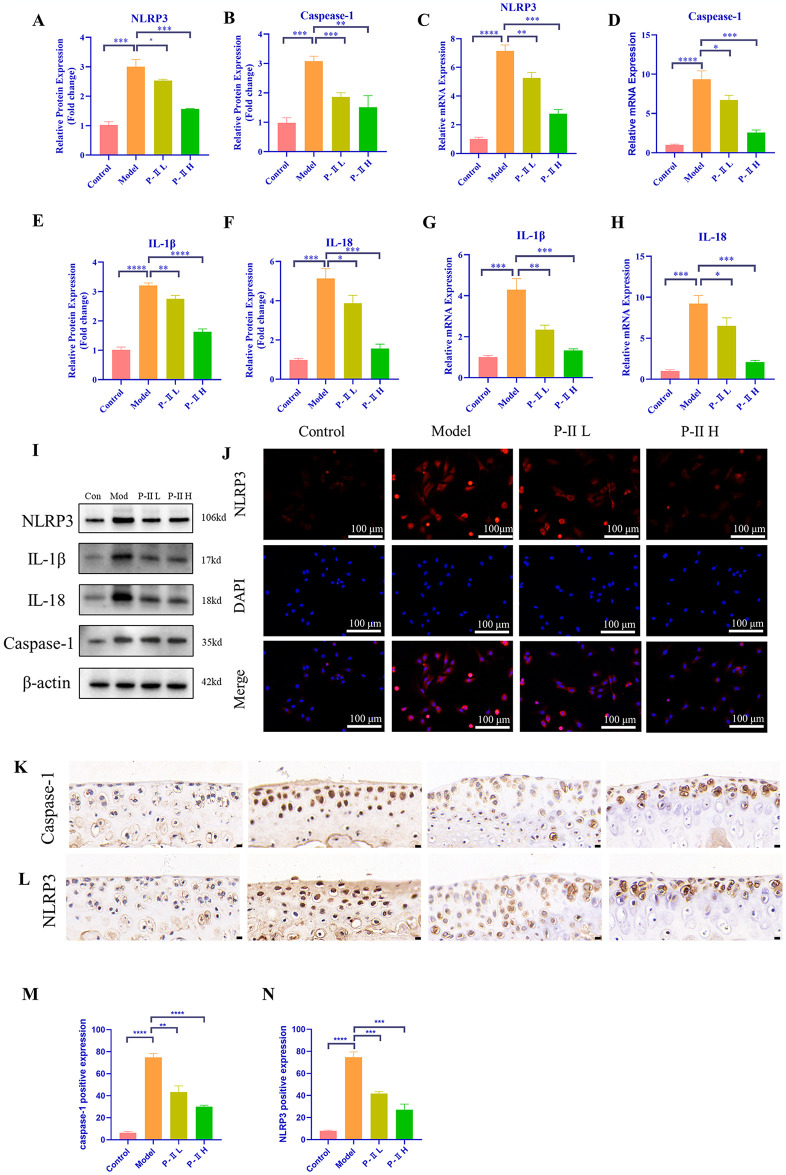
P-II suppresses NLRP3 inflammasome activation and alleviates chondrocyte pyroptosis. (A) Representative Western blot images for NLRP3, IL-1β, IL-18, caspase-1. (B-E) Quantitative analysis of the Western blot results for NLRP3, IL-1β, IL-18, caspase-1. (F-I) Quantitative analysis of qRT-PCR for *NLRP3*, *IL-1β*, *IL-18*, *caspase-1*. (J) Immunofluorescence NLRP3 (red) and dapi staining nuclear fluorescence (blue) are visible under fluorescence microscope. (K, L) Representative images of immunohistochemistry for caspase-1 and NLRP3(Scale bar = 25μm). (M, N) Quantitative analysis of caspase-1 and NLRP3 immunohistochemistry. (Data are presented as mean ± standard deviation (SD). * *P* < 0.05, ** *P* < 0.01, *** *P* < 0.001, **** *P* < 0.0001, ns signifies no statistical significance.

Pyroptosis is a type of inflammatory cell death in which the nucleus remains intact and is initiated by the NLRP3 inflammasome to activate caspase-1, releasing large amounts of the inflammatory cytokines IL-18 and IL-1β. Based on our in vitro results, we found that compared with controls, LPS-induced cells increased pyroptosis, characterized by elevated expression of NLRP3, caspase-1, IL-1β and IL-18 (Fig 3A–3I), and likewise, immunofluorescence of the cells confirmed the changes in the expression of NLRP3(Fig 3J). However, the treatment of P-II decreased their levels. In the in vivo experiments, immunohistochemical results showed that the expression of NLRP3 and caspase-1 was consistent with the changes in the in vitro experiments. Likewise, P-II decreased the expression of NLRP3 and caspase-1 in the chondrocytes of the knee joints of mice compared to the model group (Fig 3K–3N), Drawing on the findings from both in vivo and in vitro studies, it can be concluded that Picroside II treatment mitigates chondrocyte pyroptosis.

### P-II alleviates DMM-induced OA in mics

**Fig 4:**
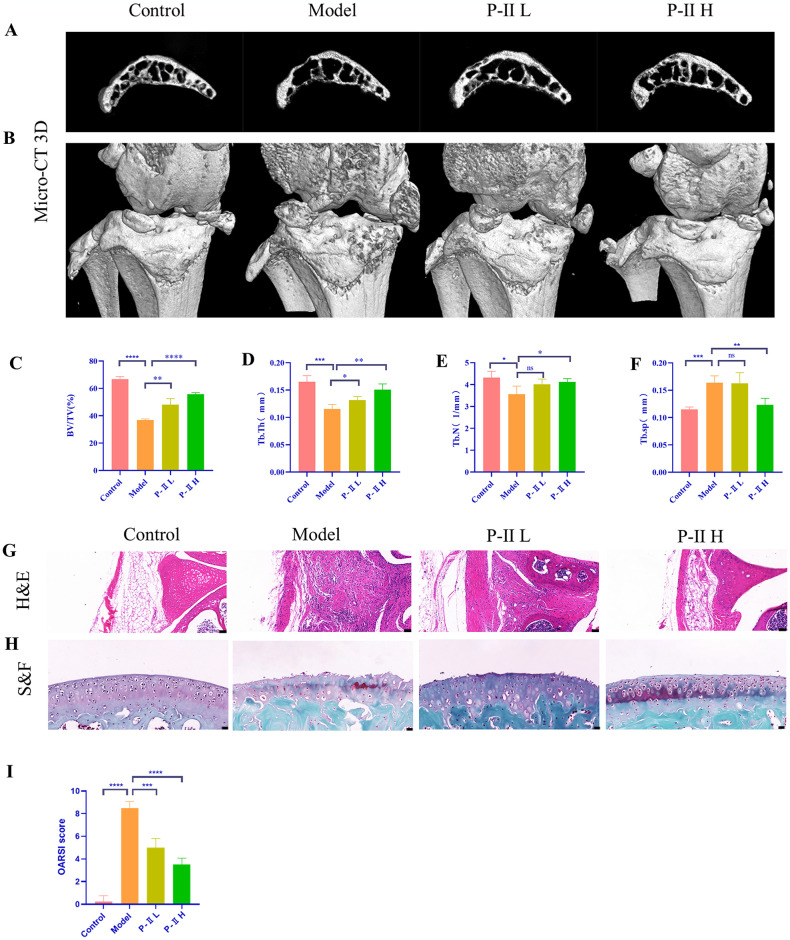
P-II reduced osteophyte formation, attenuated bone destruction, and attenuated DMM-induced OA in mice. (A). Representative Micro-CT 3D images of mice subchondral bone. (B) Representative Micro-CT 3D images of mice knee joint. (C-F) Quantitative analysis of BV/TV, Tb.Th, Tb.N, Tb.Sp of subchondral bone.(G, H) Representative images of H&E staining (Scale bar = 50μm), Safranin-O/Fast Green staining (Scale bar = 20μm). (I) OARSI score. Data are presented as mean ± standard deviation (SD). * *P* < 0.05, ** *P* < 0.01, *** *P* < 0.001, **** *P* < 0.0001, ns signifies no statistical significance.

Osteoarthritis (OA) is characterized by an inflammatory response, cartilage degeneration, and formation of osteophyte. Analysis of micro-CT scans of mouse tibiae showed that the model group had increased periarticular bone formation and greater loss of subchondral bone mass compared to the control group (Fig 4A and 4B), as well as treatment with different doses of P-II improved bone destruction in the subchondral bone. Quantitative histologic analyses showed that P-II improved BV/TV and increased Tb.Th and Tb.N, while decreasing Tb.Sp (Fig 4C–4F). These results suggest that P-II attenuates bone destruction in subchondral bone. Additionally, we found that the P-II H group was more effective than the P-II L group, and the bone reconstruction of the subchondral bone was more pronounced. In the model group compared to the control group, H&E staining in the model group showed increased synovial hyperplasia and inflammatory cell infiltration, with a higher degree of surface destruction of cartilage. After P-II treatment, There was less synovial hyperplasia in the joints and lower levels of synovial inflammation, along with better surface continuity of the cartilage than before. (Fig 4G). S&F staining showed that the model group exhibited poor articular surface integrity and uneven contours. The P-II treatment resulted in less cartilage erosion of the articular surface and more complete contour (Fig 4H). Additionally, according to the OARSI scoring system, we found that OA scores were reduced in mice in the P-II treatment group (Fig 4I), indicating that P-II can mitigate the progression of DMM-induced OA.

All these results suggested that P-II plays a positive role in improving the construction of the subchondral bone in the progression of OA.

### P-II inhibition of the MAPK/NF-κB signaling pathway

**Fig 5:**
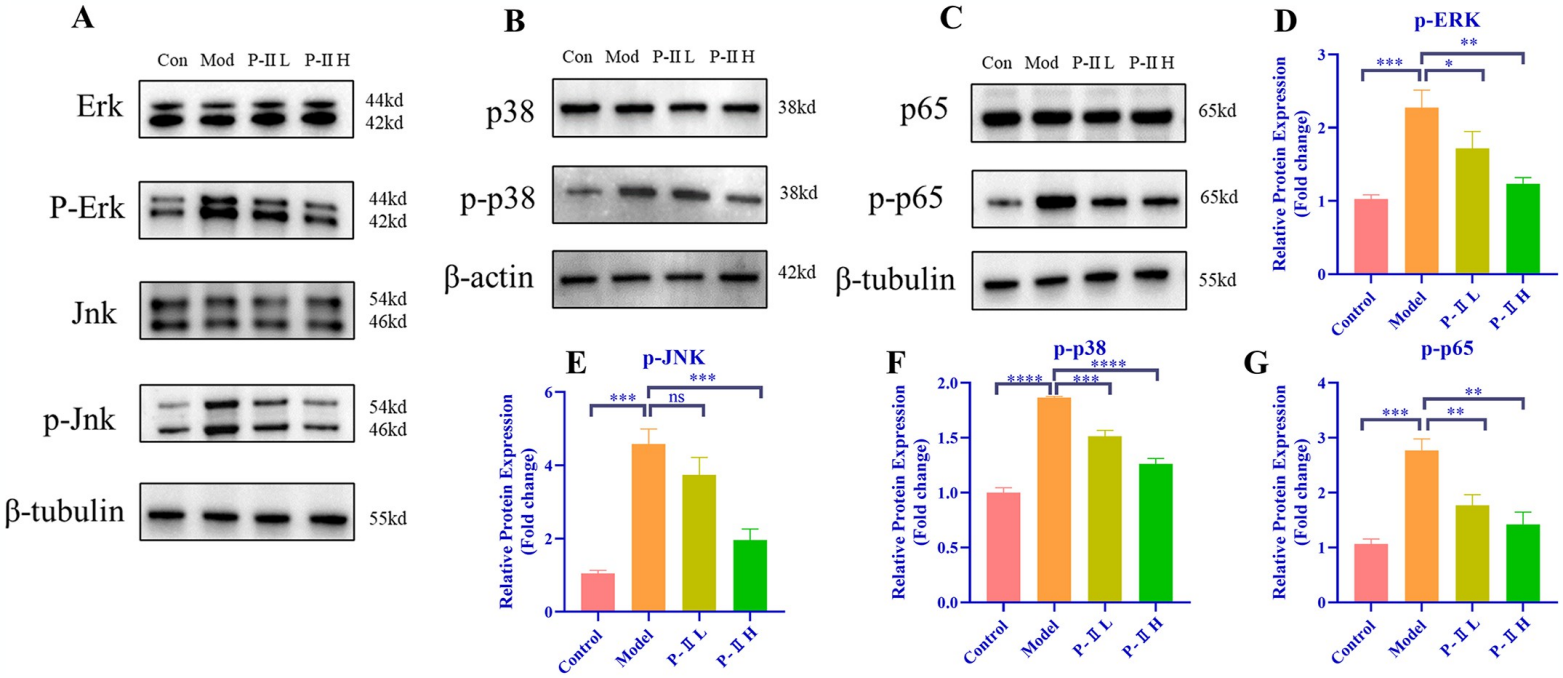
P-II inhibition of the MAPK/NF-κB signaling pathway. (A-G) Representative Western blot images for ERK, p-ERK, JNK, p-JNK, P38, p-P38, p65, p-p65 and quantitative analysis. Data are presented as mean ± standard deviation (SD). * *P* < 0.05, ** *P* < 0.01, *** *P* < 0.001, **** *P* < 0.0001, ns signifies no statistical significance.

MAPK pathway is an essential cellular signaling mediators that have been shown to lead to the activation of NLRP3 and caspase-1 [44]. Additionally, the translocation of p65 to the nucleus is a crucial step in the activation of the NF-κB pathway and is also implicated in the initiation of NLRP3 inflammasome. In this study, LPS significantly increased the phosphorylation of JNK, ERK, p38, and p65 (Fig 5A–5G) in the MAPK/NF-κB pathway. However, treatment at 25μM and 50μM of P-II significantly reduced the phosphorylation levels of these proteins. These results indicated that P-II inhibited NLRP3 inflammasome activation and mitigated inflammation by blocking the MAPK/NF-κB signaling pathway.

## Discussion

Osteoarthritis (OA) is a prevalent condition, with statistics indicating over 300 million sufferers globally. Inflammation is recognized as a central pathogenic mechanism in OA [45]. Anti-inflammatory strategies continue to be a mainstay in OA treatment [46]. Furthermore, inflammation is inextricably linked to pyroptosis. Therefore, focusing on chondrocyte pyroptosis and exploring new drugs and therapeutic mechanisms is an important research direction in OA.

Articular cartilage is predominantly made up of ECM components and is typically devoid of direct vascular or neural connections [47], Col2 is a crucial constituent of the ECM. In OA, the degradation of Col2 and the subsequent breakdown of the ECM contribute to the structural degeneration of cartilage. Meanwhile, MMP3 is an enzyme that plays a significant role in ECM degradation and is intimately connected with inflammatory processes [48]. Ma et al. noted a trend toward degradation of cartilage ECM during the progression of OA, that is to say, a decrease in Col2 expression and an increase in MMP3 expression [49]. In our study, we used 1μg /mL LPS to establish an inflammation model. Then, we used Western blot, qRT-PCR, and immunofluorescence to examine the alterations in the expression levels of Col2 and MMP3 within chondrocytes stimulated by LPS, and it is gratifying to note that our conclusions are in agreement with the previous studies, Col2 expression was decreased, while MMP3 expression was elevated in LPS-induced chondrocytes. At the same time, we found that P-II treatment increased Col2 and decreased MMP3 expression in chondrocytes, suggesting that P-II reduced ECM degradation, resulting in better preservation of the cartilage matrix. Similarly, according to the in vivo experiments, we found that immunofluorescence in knee joint chondrocytes verified the previous results, indicating that suggests that P-II reduces the degradation of ECM during OA development.

Previous studies have indicated that chondrocyte pyroptosis is an important factor in the progression of OA [50]. The classical pathway of cellular pyroptosis is the caspase-1 pathway, which recruits and activates cysteinyl asparagin-1 through NLRP3 inflammasome sensing the danger, which cleaves and activates inflammatory factors such as IL-18, IL-1β and cleaves the N-terminal sequences of the GSDMD so that it binds to the membrane and forms a membrane pore, leading to cellular pyroptosis [51]. In addition, Shen et al. showed that that P-II can attenuate the inflammatory response by inhibiting NLRP3 inflammasome and the NF-κB signaling pathway [52]. Direito, R. et al. noted that P-II exhibits potential to inhibit caspase-1 expression [53]. Therefore, we hypothesized that P-II might alleviate the progression of OA by inhibiting chondrocyte pyroptosis, and based on this inference, we conducted a comprehensive suite of validation experiments. In in vitro experiments, LPS enhanced the expression of IL-1β, IL-18, NLRP3, and caspase-1 in chondrocytes, as we expected, P-II attenuated this response. In vivo experiments, we found that the expression of caspase-1 and NLRP3 was significantly elevated in IHC of bone tissues, and reassuringly, P-II again inhibited their high expression. These results suggested that P-II can inhibit the activation of NLRP3 inflammasome in vivo and in vitro, and that it can inhibit pyroptosis by suppressing the expression of caspase-1, thereby alleviating the development of OA.

The differentiation of the roles of the MAPK and NF-κB signaling pathways in regulating chondrocyte pyroptosis is a complex molecular biology issue. The MAPK signaling pathway is typically associated with the cell’s stress response and the promotion of pyroptosis, while the NF-κB pathway is generally related to cell survival and anti-inflammatory responses. In previous studies, the MAPK signaling pathway is acknowledged for its pivotal role in regulating the proliferation and differentiation of inflammatory cells [54], and it also plays an instrumental role in cartilage differentiation and degradation. The ERK, JNK, and p38/MAPK pathways are particularly noteworthy [55]. The study by Zhou et al. states that phosphorylation of JNK/ERK/p38 leads to the development of inflammation and decreased chondrocyte proliferation [28]. And previous explorations by Lee, K et al. found that P-II has the ability to alleviate inflammation by inhibiting the MAPK signaling pathway [56]. Consequently, we investigated whether P-II could inhibit the MAPK signaling pathway and thus alleviate OA. Additionally, our experiments also investigated changes in the NF-κB signaling pathway. NF-κB is a transcription factor that regulates the immune response and inflammatory response, and regulates gene expression in response to multiple stimuli [57], and is a key signaling molecule for NLRP3 inflammasome activation [58]. P-II has demonstrated the ability to suppress LPS-induced inflammation by targeting the NF-κB signaling pathway, where p65 phosphorylation is a critical step in activation [59,60], p65 phosphorylation is activated into the nucleus and promotes the expression of a range of inflammation-related genes55. We therefore investigated whether P-II could inhibit the phosphorylation of p65, p38, JNK, ERK. Happily, our experimental results showed that phosphorylation of p65, p38, ERK, JNK was significantly elevated in response to LPS induction, but was reversed by P-II, consistent with previous studies, suggests that P-II has a role in inhibiting the MAPK/NF-κB signaling pathway. This further confirms our hypothesis and suggests that P-II may ameliorate chondrocyte pyroptosis through the MAPK/NF-κB pathway.

## Conclusion

**Fig 6:**
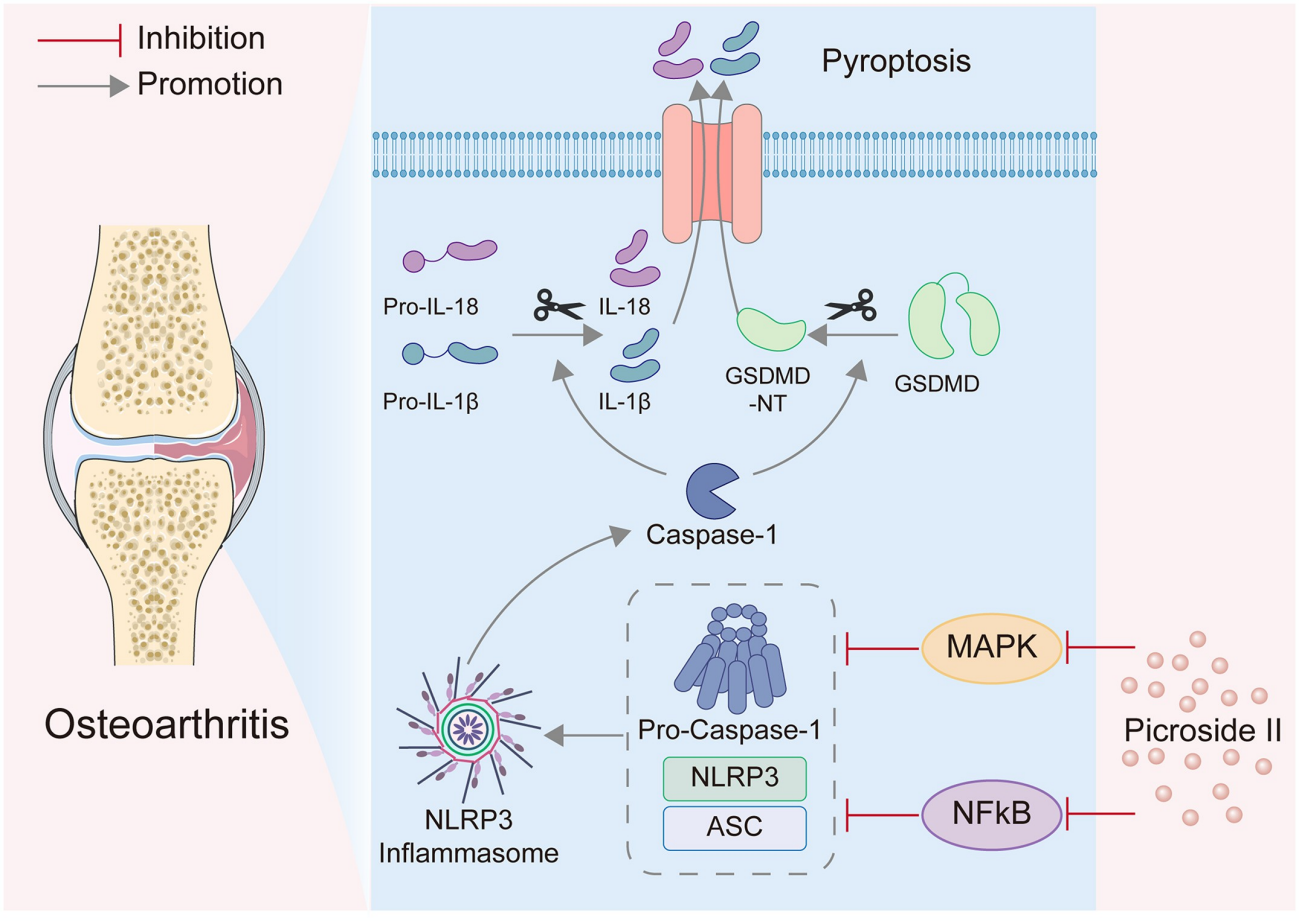
Potential mechanism of action of Picroside II in osteoarthritis.

In conclusion, we identified for the first time that P-II has a therapeutic effect on osteoarthritis, and the underlying molecular mechanism may be achieved by attenuating chondrocyte pyroptosis through inhibition of the MAPK/NF-κB pathway. Therefore, targeting NLRP3 inflammasome by P-II may be a potential strategy for the treatment of OA (Fig 6). The modulatory effects of P-II on chondrocyte NLRP3 inflammasome activation and pyroptosis and its safety evaluation for clinical application deserve in-depth study.

## Supporting information

S1 Raw image (PDF)

S1 Raw data (ZIP)

## Abbreviations

BV/TV: Bone volume / tissue volume
CCK-8: The Cell Counting Kit 8
DMM: destabilization of the medial meniscus
ECM: Extracellular Matrix
EDTA: Ethylene Diamine Tetraacetic Acid
H&E: Hematoxylin-Eosin
OA: Osteoarthritis
OARSI: Osteoarthritis Research Society International
S&F: Safranin O-Fast Green
Tb.N: Number of trabeculae
Tb.Sp: Trabecular separation
Tb.Th: Trabecular thickness
