# Supplementary material for: Picroside II suppresses chondrocyte pyroptosis through MAPK/NF-κB/NLRP3 signaling pathway alleviates osteoarthritis
Source: PLoS One. 2024 Aug 29;19(8):e0308731. doi: 10.1371/journal.pone.0308731 (PMC11361613; doi:10.1371/journal.pone.0308731)
Supplement: S1 Raw image — (PDF) [file pone.0308731.s001.pdf]

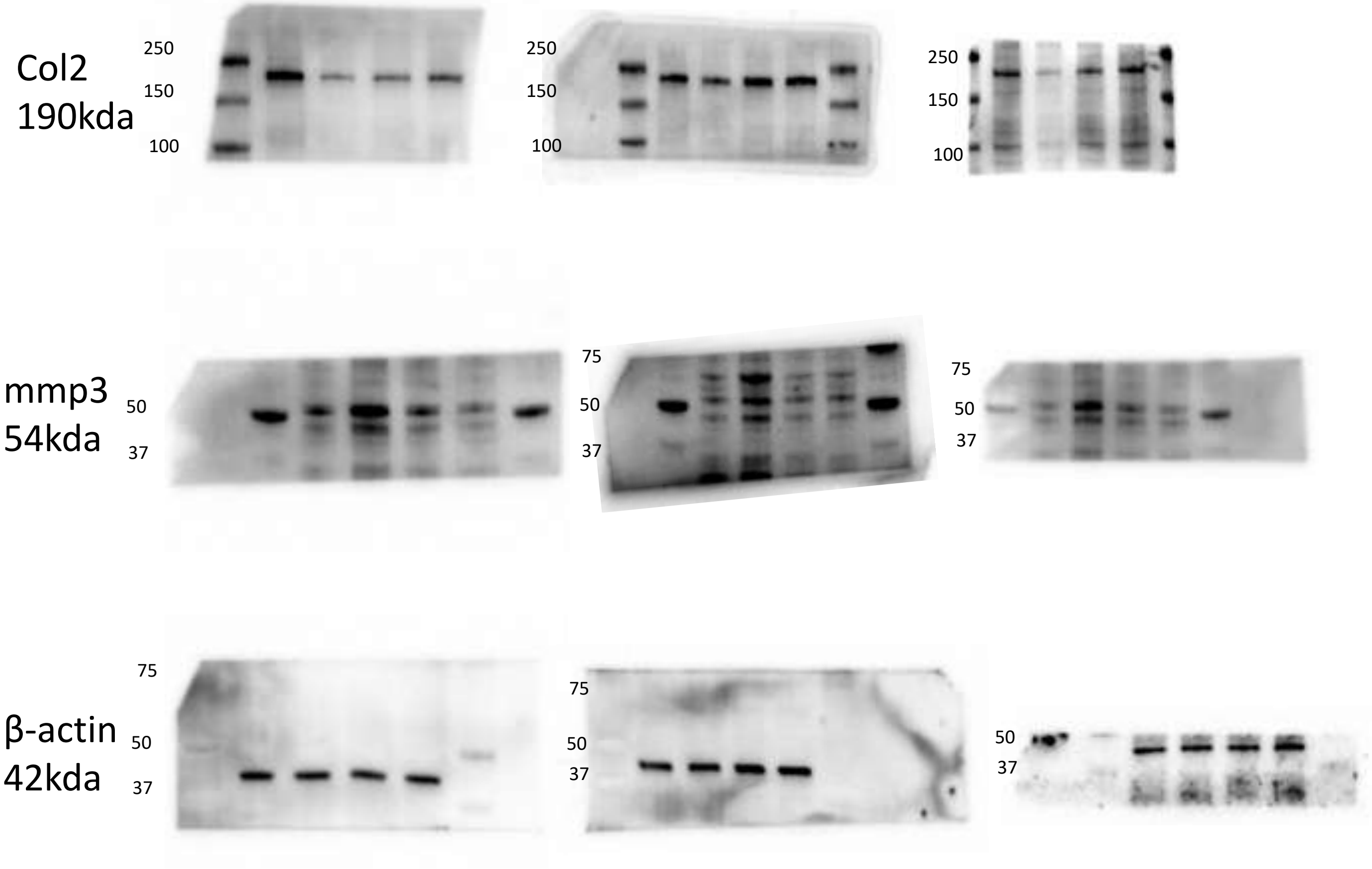
Col2
190kda
250
150
100
250
150
100
250
150
100
mmp3
54kda
50
37
75
50
37
75
50
37
β-actin
42kda
75
50
37
75
50
37
50
37

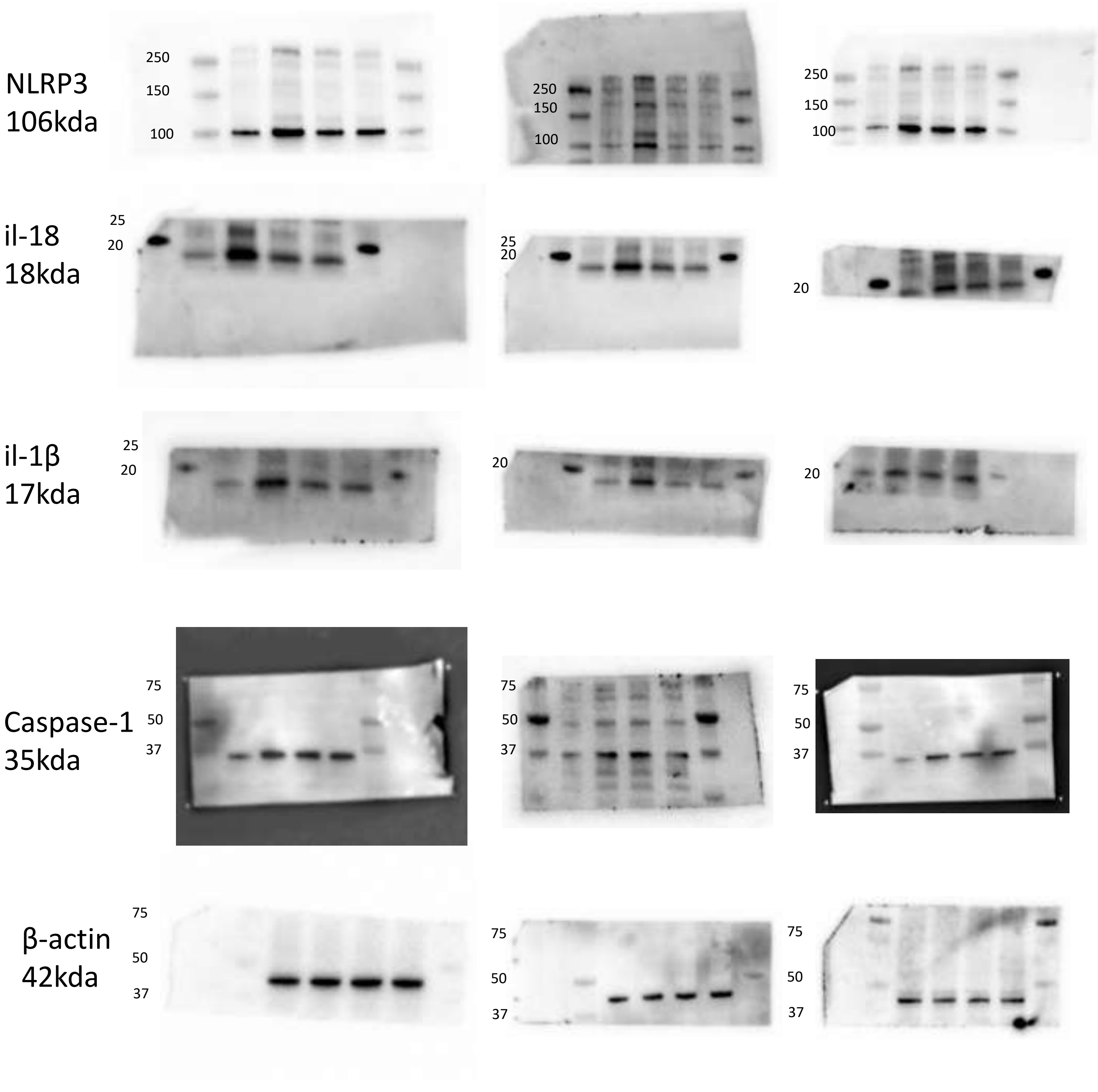
NLRP3
106kda
250
150
100
il-18
18kda
25
20
il-1β
17kda
25
20
Caspase-1
35kda
75
50
37
β-actin
42kda
75
50
37

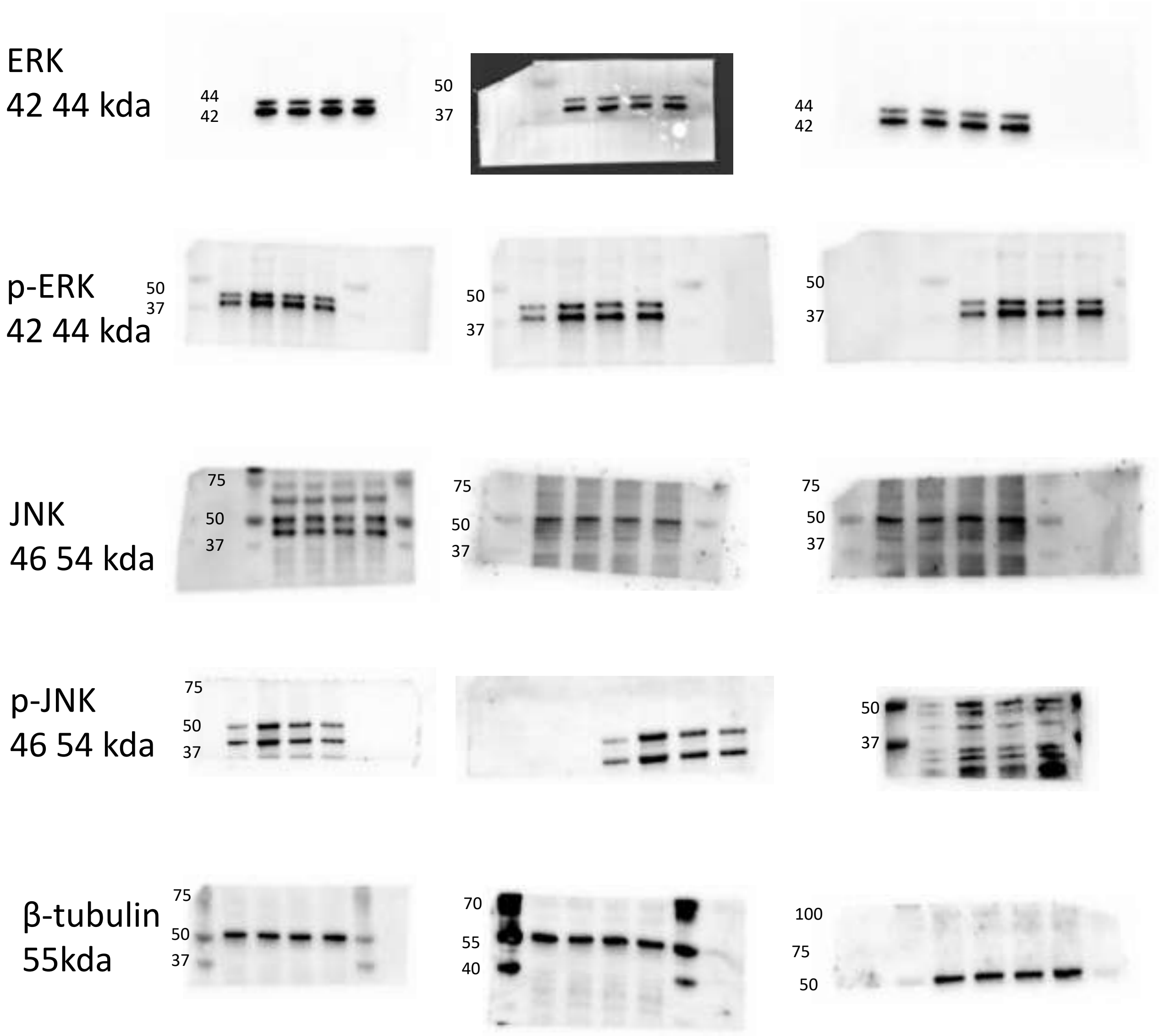
ERK
42 44 kda
44
42
50
37
44
42
p-ERK
42 44 kda
50
37
50
37
50
37
JNK
46 54 kda
75
50
37
75
50
37
75
50
37
p-JNK
46 54 kda
75
50
37
50
37
β-tubulin
55kda
75
50
37
70
55
40
100
75
50

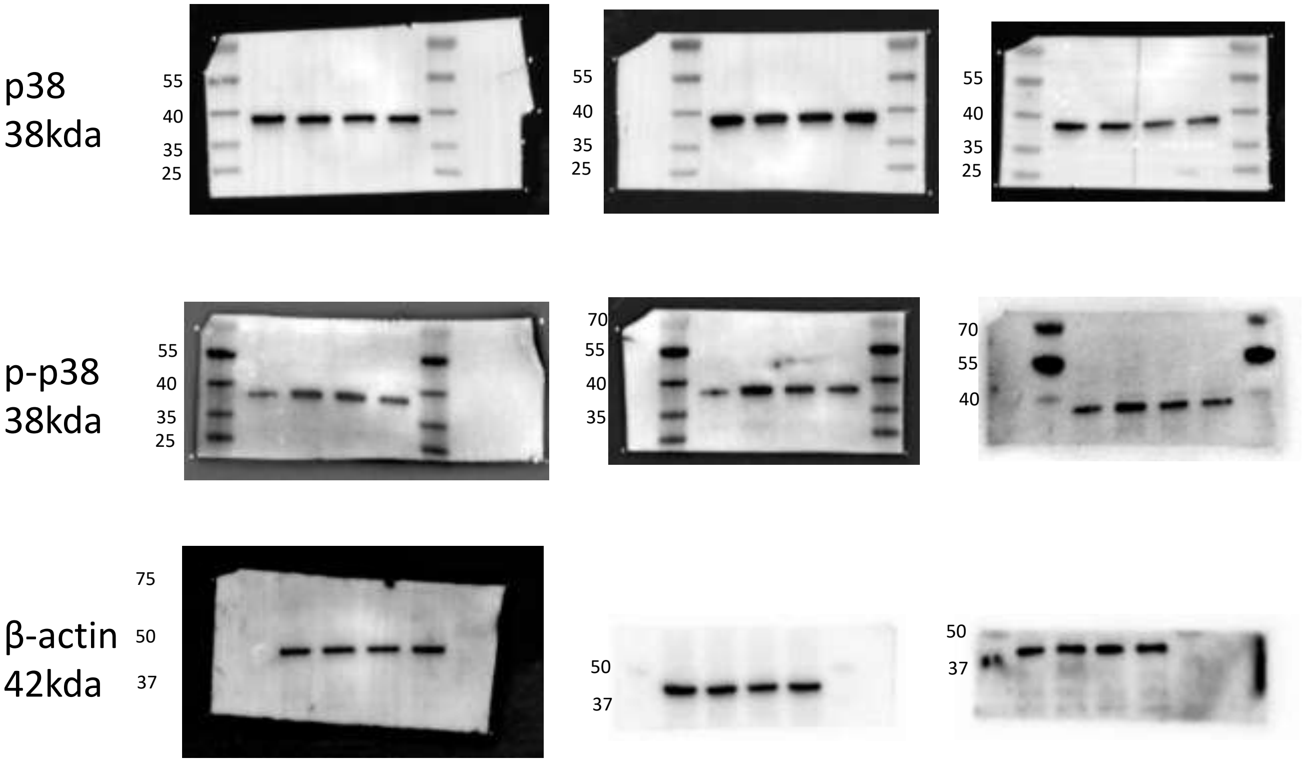
p38
38kda
55
40
35
25
55
40
35
25
55
40
35
25
p-p38
38kda
55
40
35
25
70
55
40
35
70
55
40
β-actin
42kda
75
50
37
50
37
50
37

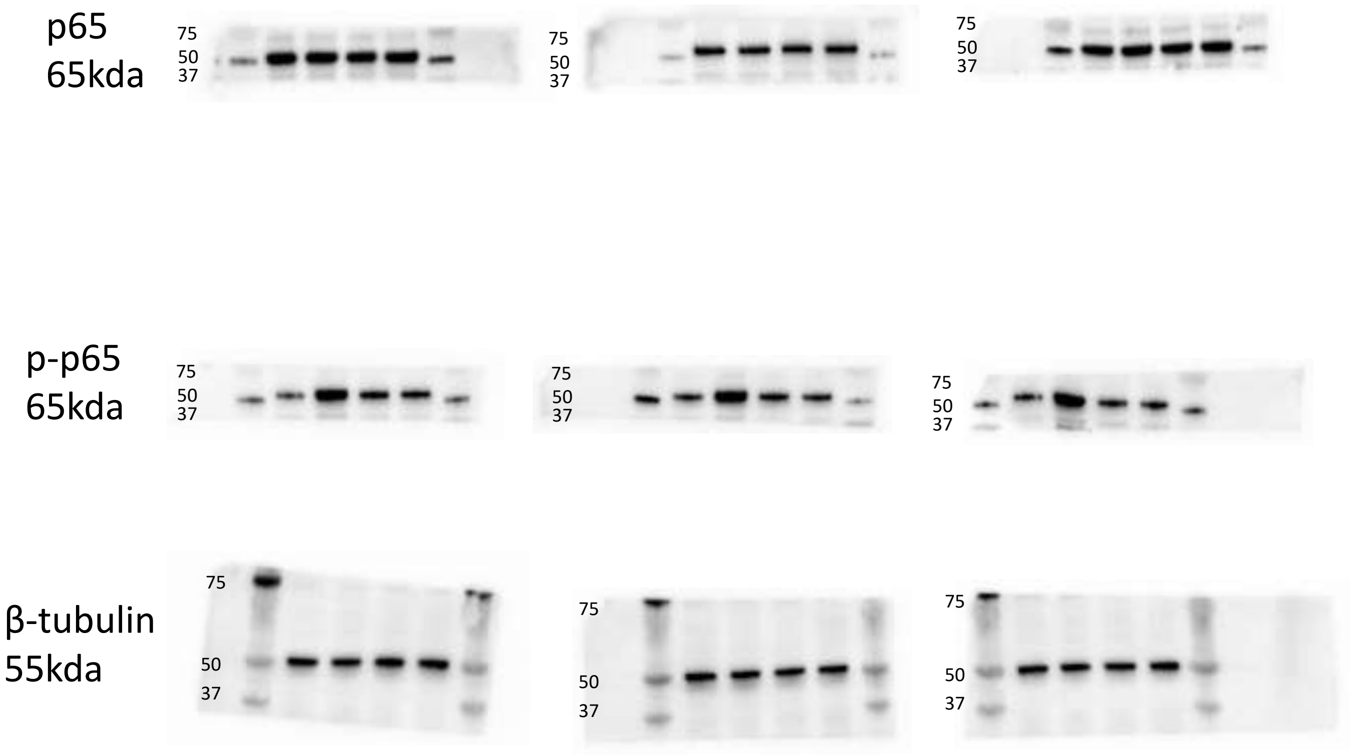
p65
65kda
75
50
37
p-p65
65kda
β-tubulin
55kda
